# Rapid Determination of Ochratoxin A in Grape and Its Commodities Based on a Label-Free Impedimetric Aptasensor Constructed by Layer-by-Layer Self-Assembly

**DOI:** 10.3390/toxins11020071

**Published:** 2019-01-28

**Authors:** Mina Nan, Yang Bi, Huali Xue, Sulin Xue, Haitao Long, Lumei Pu, Guorui Fu

**Affiliations:** 1 College of Horticulture, Gansu Agricultural University, Lanzhou 730070, China; nanmn@gsau.edu.cn (M.N.); xuesulin@outlook.com (S.X.); 2 College of Food Science and Engineering, Gansu Agricultural University, Lanzhou 730070, China; 3 College of Science, Gansu Agricultural University, Lanzhou 730070, China; longht@gsau.edu.cn (H.L.); pulm@gsau.edu.cn (L.P.); fugr@gsau.edu.cn (G.F.)

**Keywords:** grape, ochratoxin A, thiolated aptamer, label-free, layer-by-layer self-assembly

## Abstract

A simple and sensitive label-free impedimetric aptasensor for rapid determination of ochratoxin A (OTA) has been developed, which was based on the combination between thiolated aptamer and gold nanoparticles by layer-by-layer self-assembly. Because of the interaction between aptamer and OTA, the relative normalized electron-transfer resistance (ΔR_ct_) values obtained by electrochemical impedance spectroscopy (EIS) was proportional to the concentration of OTA and showed a good linear relationship from 0.1 to 10.0 ng/mL, with a lower detection limit (0.030 ng/mL) than one-step thiolated DNA aptasensor. The established method was successfully applied to detect and analyze OTA in table wine and grape juice, and the recovery was 90.56%–104.21% when PVP effective removed of phenolic substances. The label-free impedimetric aptasensor was used for rapid detection and quantitation of OTA in the inoculated grapes with the *Aspergillus Nigri* (H1), and the production of OTA (62.4 μg/kg, 20 μg/kg) far exceeded the maximum levels of 2 μg/kg after inoculation for three days. The developed method exhibited a good specificity, high sensitivity, time-efficient, and it could be applied to detect the OTA concentration in grape and its commodities.

## 1. Introduction

Grape is one of the world’s largest fruit crops with the highest proportion of processing, such as wine, grape juice, raisins, and other products. Grapes are easily infected by various fungi, which result in the grape decay and mycotoxins contamination [1,2]. *Aspergillus* and *Penicillium* are major agents infected grape at the stage of pre-harvest and post-harvest, ochratoxin production occurs during this time and the accumulation of toxins increases rapidly in fruit ripening [3,4,5]. Ochratoxin is a group of important mycotoxins, which mainly contain three important members with similar structures [6,7]. Among them, ochratoxin A (OTA) is the most widespread and toxic in nature, with a variety of subchronic and chronic toxicity to human, such as nephrotoxicity, neurotoxicity, teratogenicity, immunotoxicity, mutagenicity, and hepatotoxicity [8,9,10]. In view of OTA contamination and toxic threat, it is essential to develop a simple and rapid OTA detection method with high selectivity and accuracy. 

The conventional methods of detecting OTA depend on high performance liquid chromatography (HPLC) combined with fluorescence detection (FD) [11] or mass spectrometry detection (MS) [12,13], or gas chromatography (GC) [14]. However, all these methods have the limitation of low specificity, long and expensive detection process, and demand further purification of sample before analyzing [15]. In view of the high specificity and affinity of the antigen-antibody reaction, immunochemical methods become paramount importance in most tests of OTA determination, such as enzyme-linked immunosorbent assay (ELISA) [16,17] and fluorescence polarization immunoassays [18]. Although immunological-based methods are user-friendly and have good sensitivity, these methods often suffer from being time-consuming, difficulty to modify, poor stability of antibodies, and batch to batch variation differences. Biochemical sensors based on aptamer have been proved to be an alternative method for rapid detecting of OTA [19,20,21,22]—for example, aptasensor modified with Au-ATP-rGO composite [20], polythionine (PTH) and iridium oxide nanoparticles (IrO2 NPs) [21], and azido-aptamer onto electrografted binary film via click chemistry [22]. Note that most of these aptasensor with good detection limit and linear detection range require expensive materials and specific chemical reactions. Immobilization of thiolated aptamer by layer-by-layer self-assembly could achieved the equal effect. More importantly, it is low-cost and easier to be obtained. Moreover, although these sensors were applied to determine OTA in wine [20,21], the application of biochemical sensor for determining of OTA in *Aspergillus niger*–infected grapes has not been reported.

Electrochemical aptamer biosensor is a sensor with a DNA or RNA aptamer as the identification element. Because of the high sensitivity and selectivity, fast response, simple operation, low cost, and miniaturization of electrochemical aptasensor, it is widely researched in mycotoxin inspection [19]. Based on whether the detection signals are produced by markers, the electrochemical aptamer sensors are divided into two categories—labeled and labeled-free. Although the labeled electrochemical aptamer sensor has high sensitivity, its development is limited because of its complex construction and high cost [23]. In order to avoid the using of complex and expensive labeling markers, the labeled-free electrochemical aptasensor for OTA detection was developed [24,25,26,27]. However, how to improve the poor stability of aptamer fixation, simplify the preparation process, reduce the use of toxic chemicals, and increase the sensitivity of detection signals are crucial problems in the process of constructing the labeled-free aptamer sensor [27].

Layer-by-layer self-assembly is one of the simplest methods to immobilize the biomolecules on a sensor [28]. It is easy to obtain and well oriented, resulting in good activity of biomass being maintained [29]. Self-assembled monolayers (SAMs) are molecules ordered in the solution that are strongly adsorbed on solid substrates by covalent bonds, electrostatic attraction, and hydrogen bonds [30]. Therefore, based on the method of layer-by-layer self-assembly, the aptamer can be easily fixed, and the fabrication process of the sensor will be greatly simplified without multiple chemical reactions. 

In this study, based on self-assembled monolayers of thiolated aptamer on gold substrates, the label-free impedimetric aptamer sensor was constructed. Gold nanoparticles and the thiol-groups formed the strong S-Au bond, which kept the label-free aptamer sensor stable. Gold nanoparticles modified the electrode, which had a large surface area for DNA immobilization [31], that was used as signal amplifying equipment. This sensor was applied to detect the concentrations of OTA, and it was proved that it is a simple and fast method with high selective and sensitive and reached good integrative effects. OTA in grape products with PVP pretreatments were studied to improve the accuracy of detection. To our knowledge, for the first time the concentrations of OTA in grapes infected with *Aspergillus niger* were detected by a label-free aptasensor.

## 2. Results

### 2.1. Principle and Characterization of Label-Free Impedimetric Aptasensor

**Scheme 1:**
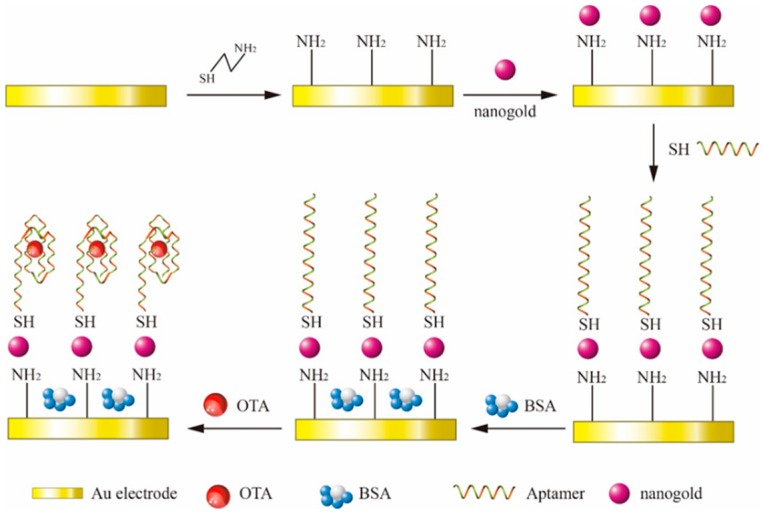
Schematic presentation of the fabrication steps and principle of the the label-free impedimetric aptasensor.

The proposed sensor used in this study is based on the principle of layer-by-layer self-assembly, and the detection applied [Fe(CN)_6_]^3−/4−^ as a redox probe. The mechanism of the label-free impedimetric aptasensor was illustrated in Scheme 1. First, the sulfhydryl group of Cysteamine (CA) molecule forms chemical bonds with bare gold electrode to form monolayer self-assembled film. After that, gold nanoparticles were immobilized on the CA modified electrode surface through electrostatic interactions between the negatively charged citrate radicals around it and the positively charged amino-group of CA. Gold nanoparticles with larger surface area than bare gold electrode can immobilize more aptamer [31]. The sulfhydryl group aptamer combined with gold nanoparticles by Au-S bond. The aptamer self-assembled monolayers were negative charged because of the exposition of bases caused by aptamer uncoil [32], which directly result in the electrostatic repulsion between aptamer and redox probe. Because there were gaps between the modified molecule, BSA was used to completely block the nonspecific adsorption sites to avoid the electrical signal produced by the diffusion of [Fe(CN)_6_]^3−/4−^ to the unmodified gold electrode. After BSA modified step, the label-free impedimetric aptasensor was prepared and was used for the determination of OTA. OTA showed weak acidic and always negative charged as the form of OTA^−^ and OTA^2−^ at neutral pH [33]. With addition of OTA^−^ and OTA^2−^, the electrostatic hindrance would increase due to increased negative charge of the sensor. In addition, once the OTA binding to the aptamer, the antiparallel G-quadruplex conformation could be formed from random coil structure [32]. This will greatly increase the steric resistance of the transfer of the redox probe. The more OTA the sensor combined, the higher electron transfer resistance.

**Figure 1:**
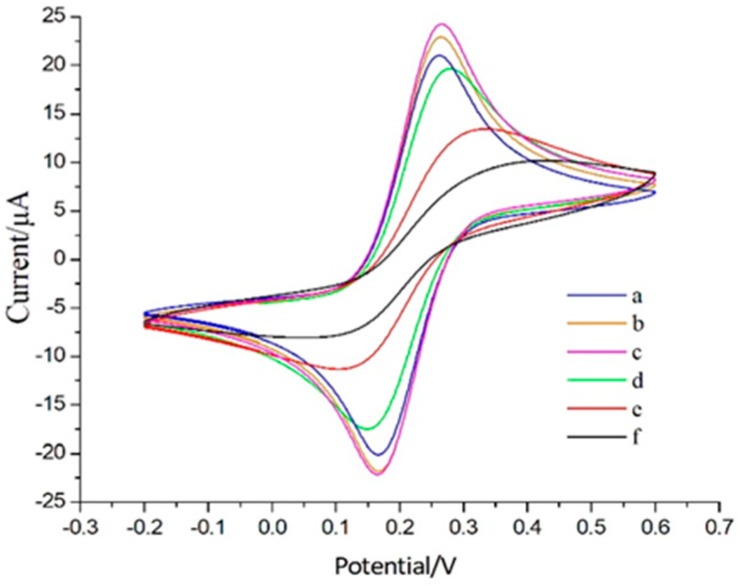
Cyclic voltammograms of each modified step of aptasensor in 0.5 mM [Fe(CN)_6_]^3−/4−^ in 10 mM KCl solution, with a scan rate of 50 mV/s; (a) bare Au; (b) CA/Au; (c) AuNPs/CA/Au; (d) aptamer/AuNPs/CA/Au; (e) BSA/aptamer/ AuNPs/CA/Au; (f) OTA/BSA aptamer/ AuNPs/CA/Au.

The interface properties of surface modified aptamer sensor were characterized by CV. Figure 1 shows the cyclic voltammetric curves of each fabrication step of label-free impedimetric aptasensor. At the bare gold electrode, the redox probe presented a good reversible voltampere curve (curve a), indicating that the electron transfer was completely controlled by the diffusion of molecules. As the CA molecule is assembled on the surface of the gold electrode, the N atoms in CA can combine with H^+^ to form positive ions. The surface of the CA monolayer with high positive charge has strong electrostatic attraction effect on [Fe(CN)_6_]^3−/4−^. Thus, redox probe in the solution was easy to adsorb and accumulate on the electrode surface, resulting in current enhancement. The CA-modified electrode soaked in gold nanoparticles solution and the redox peak current increased (curve c). It proved that the gold nanoparticles formed a new gold electrode surface increasing the surface area [31]. After the formation of sulfhydryl group aptamer self-assembled monolayers, the diffusion of the redox probe into the surface of the electrode was hindered, so the peak current was reduced (curve d). Then the peak current decreased accordingly with the immobilization of BSA (curve e). In the determination of OTA, peak current lowed down, while the peak-to-peak separation increased. This result showed that the electron transfer of redox probe was hampered both by electrostatic and steric repulsion. All the results mentioned above demonstrated the feasible principle and the successful fabrication of the sensor. This sensor could be regenerated by immersing it in 1 mM HCl for 30 s after each OTA determination [34,35]. 

### 2.2. The Optimization of Fabrication for Label-Free Impedimetric Aptasensor

In the process of sensor preparation, the formation time of monolayer is crucial to the performance of sensor. The preparation conditions were screened and optimized to obtain the best stability of the sensor. The modification time of CA was 24 h, while the peak current decreased when the modified time was too long. The CA modified gold electrode was soaked in gold nanoscale solution. The peak current increased gradually as the reaction time increased and reached the maximum at 8 h. After 8 h, the peak current decreased because the electrode was no longer monolayer modified. The electron transfer resistance increased with incubation time extension, and the signal reached saturated at 60 min. After that the electron transfer resistance (ΔR_ct_) almost kept sable during the whole incubation time. Thus, the incubation time of OTA was selected as 60 min. 

### 2.3. Detectability of the Label-Free Impedimetric Aptasensor

#### 2.3.1. Detection of OTA by Label-Free Impedimetric Aptasensor

**Figure 2:**
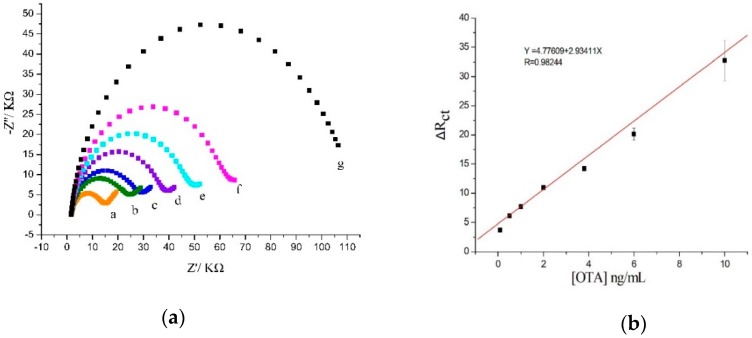
(**a**) Nyquist plot of the label-free impedimetric aptasensor with different OTA concentrations. (a–g) 0.1 ng/mL, 0.5 ng/mL, 1 ng/mL, 2 ng/mL, 3.8 ng/mL, 6 ng/mL, 10 ng/mL. (**b**) Calibration curve of the ΔR_ct_ values vs OTA concentration from 0.1 ng/mL–10 ng/mL.

Ochratoxin A was dissolved in methanol and was then diluted with binding buffer (BB pH 7.4) to get a series of concentrations (0.05 ng/mL–200 ng/mL) of OTA. EIS was applied to study the impedance changes of the sensors in different concentrations of OTA after cultivation 60 min. With the increasing of the concentration of OTA, the electron transfer resistance increased (Figure 2a). Because of the dissociation of carboxyl and the phenolic hydroxyl dihydro isocoumarin, OTA was negatively charged in the BB solution [35]. As the amount of OTA that binds aptamer increased, the electronegativity of the electrode surface increased. On the other hand, when the amount of OTA was increased, the more structure of antiparallel G-quadruplex formed, which lead to the larger space hindrance. Thus, the [Fe(CN)_6_]^3−/4−^ was more difficult to reach the electrode surface and produce the current, that is to say, the electron transfer resistance increased. 

The ΔR_ct_ of the OTA/BSA/Apt/AuNPs/CA/Au modified electrode had a linear relationship with the concentration of OTA at the concentration rang from 0.1 ng/mL to 10 ng/mL (Figure 2b). The linear equation was ΔR_ct_ = 4.77609 + 2.93411C_OTA_ (ng/mL), the linear correlation coefficient (R) was 0.9824, and the detection limit was 0.030 ng/mL, which was calculated based on the average value of background signals (2.27) and the threefold standard deviation of the background signal (0.23) [36]. LOD in this work was much lower than the maximum levels of OTA prescribed by the European Commission (EU).

#### 2.3.2. Performance Comparison with Reported Label-Free Aptasensor 

The performance of the label-free aptasensors have been reported was compared with this work (Table 1). According to the results, LOD and linear range achieved in this research showed competitive performance with other aptasensors. In addition, although most of these free-label impedimetric sensors could achieve low detection limits, the fixation of aptamer was difficult even in some methods involved complex modified material and toxic chemicals. Castillo et al. directly modified the thiolated DNA aptamers on the surface of gold electrode, the fabrication is simple; however the detection limit of this method was higher than the results in our work [35]. As we know, the layer-by-layer self-assembly to prepare the aptamer sensor in this study is one of the simplest methods for the formation of label-free impedimetric aptasensor to detect OTA. In summary, the label-free impedimetric aptasensor for OTA detection in this work is simpler and cheaper, allowing to achieve the low LOD with good detection range.

### 2.4. Specificity of Label-Free Impedimetric Aptasensor 

**Figure 3:**
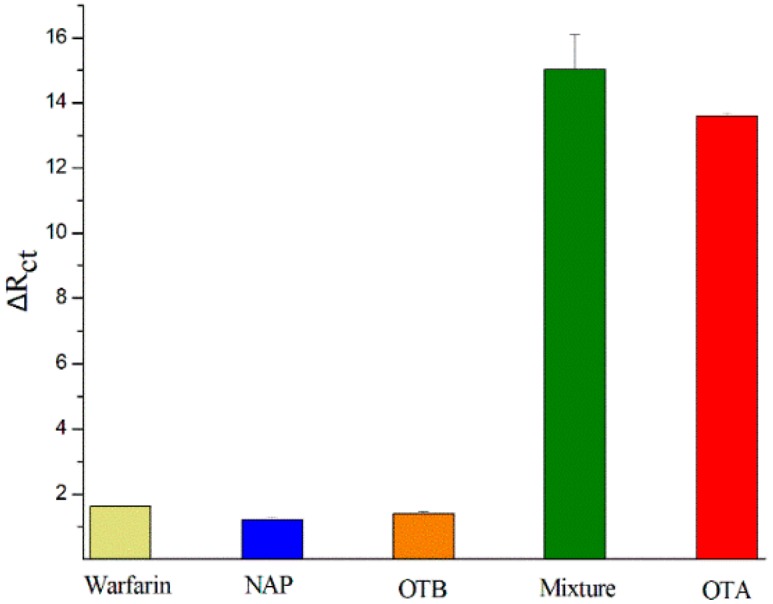
The specificity of the sensor in the presence of 10 ng/mL warfarin, NAP, OTB, OTA, mixture of the warfarin, NAP, OTB, and OTA.

Whether sensors can specifically identify target molecules and avoid interfering signals produced by structure analogues of target is of particular importance for sensor feasibility assessment. As a control experiment, three substances—ochratoxin B (OTB), N-acetyl-l-phenylalanine (NAP), and Warfarin—were selected as dummy substrates. These dummy substrates and the mixture of them with OTA were detected by the label-free aptamer sensor. The ΔR_ct_ value of the OTA at concentration of 10 ng/mL was used as the contrast standard. All the other toxins were at the same concentration and also incubated on the sensor for 60 min. Impedance responses is shown in Figure 3. The ΔR_ct_ of mixture was obviously higher than that for the other three kinds of toxins, but it closed to the value of the OTA. This was the result of the specific recognition of OTA by the aptamer and indicates that the specificity of this sensor was excellent.

### 2.5. Practicability of Label-Free Impedimetric Aptasensor

#### 2.5.1. Application of Label-Free Impedimetric Aptasensor in Grape Commodities

**Figure 4:**
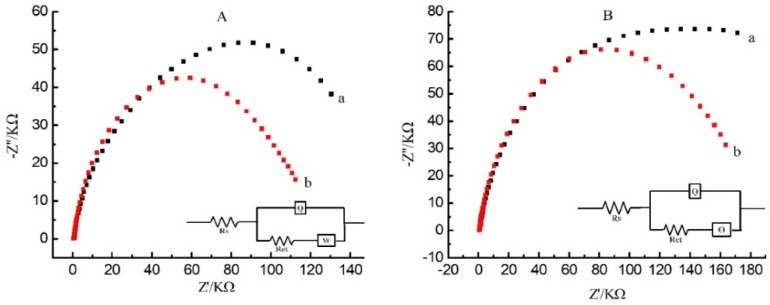
(**A**): Nyquist plot of the label-free impedimetric aptasensor with spiked white wine (a) untreated sample; (b) PVP treated samples; (**B**): Nyquist plot of the label-free impedimetric aptasensor with spiked red wine. (a) untreated sample; (b) PVP treated samples. Inset: The equivalent circuit model; A: (RQ(RW)) model; B: (RQ(RO)) model.

In order to investigate the practical application of the designed aptamer sensor, we chose red wine, white wine, red grape juice, and purple grape juice spiked with a series of concentrations of OTA to evaluate the analytical reliability. OTA was detected in untreated and PVP-treated wine. Comparing the experimental results, it was found that the impedance curves did not show a good arc in untreated wine (Figure 4). The data no longer fit with the (RQ(RW)) model but was consistent with (RQ(RO)) model, indicating that the diffusion control mode has changed from infinite diffusion to finite diffusion. The two equivalent circuits contained different impedance elements—the Warburg impedance element (W) and the Hyperbolic tangent impedance element (O). The Warburg impedance element is caused by diffusion of probe molecules, while the Hyperbolic tangent impedance element is produced by many reasons, such as the diffusion through the thin film, slow diffusion of oxygen in the passivation layer, or ion-selective electrode and so on. Because the terminal inductive impedance (low frequency region) is usually produced by the adsorption of surface reactants, we assumed that because of hydroxyl in the phenolic compounds of wine without PVP treatment, adsorption thin films would be formed on the electrode surface by hydrogen bonds, and the diffusion of probe molecules had to across the thin films and resulted in electrical abnormal impedance curves (black scatter plots).

Recovery experiments were carried out on four kinds of grape products pretreated by PVP to eliminate matrix effect. According to the results of Table 2, the recovery rates (90.56%–104.21%) of OTA in treated samples determined by this sensor were excellent. In most reports of the directly determination of OTA in wine samples by aptasensor, the recovery was not satisfactory because of the unspecific adsorptions of components of the wine matrix [21,35]. A double liquid–liquid extraction needed several times phase separation, which was very complex and time-consuming [38]. When the spiked sample were separated and purified through high-cost immune-affinity column (IAC), the recovery percentage could be better [27]. However, IAC is costly for detection. The pre-treatment of wine and grape juice by PVP was very simple, which can save the cost and efficiently removal the phenolic compounds components of the wine. This method could guarantee the detection effect and be extended to the detection of OTA by aptasensor in other fruit juice and table wine. 

#### 2.5.2. Application of Label-Free Impedimetric Aptasensor in Grape

As raw materials for table wine, the contamination degree of OTA in grapes directly affects the content of OTA in wine. Therefore, the determination of OTA production in grapes infected by fungi is particularly important. Grapes (Cv. Centennial seedless, red globe) were inoculated with the *Aspergillus niger* (H1). According to the literature, the strain H1 could produce OTA both on Yeast extract sucrose agar (YES) medium and Czapek yeast extract agar (CYA) medium, and it was proved to be one of the producing strains of OTA [39]. These grapes inoculated on different days were used as the object of detection, and OTA content was detected by the prepared sensors. The production of OTA in grapes per gram was calculated by the determined OTA concentration according to the extraction method of OTA (Table 3). The result showed that OTA were produced in different varieties of grapes after inoculation with *Aspergillus niger*, but the concentration of OTA was different. After three days of inoculation the OTA levels, 62.4 μg/kg in red globe and 20 μg/kg in centennial seedless, far exceeded the OTA maximum levels (2 μg/kg) in grape and related commodities set by European Commission (EU) [27]. After seven days of incubation on red globe and centennial seedless, the OTA production were about 200 μg/kg and 140.4 μg/kg, respectively, which were much lower than the production of OTA on YES medium (26.01 μg/g YES) [39]. On the basis of these data, the sensor was proved to be practical and effective for the determination of OTA in grapes. 

## 3. Conclusions

A simple method for the formation of aptamer sensors based on layer-by-layer self-assembled was developed in this study. The preparation of the sensor based on the self-assembled monolayer greatly simplified the construction of the sensor. The developed label-free impedimetric aptasensor was successfully applied to detect OTA, which obtained a linear relationship at a concentration range from 0.1 ng/mL–10 ng/mL, the detection limit was 0.030 ng/mL. The sensor showed excellent specificity and high sensitivity and accuracy. The preconditioning of PVP in grape samples eliminated the interference of the phenolic substances to the electrical signals. After PVP treatment of red wine, white wine, and grape juice, a good recovery range (90.56%–104.21%) was obtained. The detection results of grapes showed that OTA can be produced by the strain H1 of *Aspergillus* section *Nigri* when it infects grapes. After three days of inoculation, the production of OTA (62.4 μg/kg, 20 μg/kg) far exceeded the EU maximum limit of 2 μg/kg. By replacing the aptamer sequence in the sensor, this method could be also applicable to other aptamers and the detection of their corresponding substances.

## 4. Materials and Methods

### 4.1. Chemical Reagents

The thiolated aptamer was synthesized by Synbio Technologies (Shanghai, China), the sequence of the aptamer was shown below: 5′-GATCGGGTGT GGGTGGCGTA AAGGGAGCAT CGGACA-3′, 5′-SH. OTA and OTB (analytical standard) used in this study was purchased from Pribolab Pte. Ltd. (Qingdao, China). Gold particles were synthesized from Chloroauric acid (HAuCl_4_) and sodium citrate according to the literature [40]. 

Cysteamine (CA), Bovine serum protein (BSA), N-acetyl-l-phenylalanine (NAP), and Warfarin were purchased from Shanghai Yuanye Bio-technology Co. Ltd. (Shanghai, China). Potassium ferrocyanide (K_4_[Fe(CN)_6_]) and, potassium ferricyanide (K_3_[Fe(CN)_6_]), were purchased from Sinopharm Chemical Reagent Co. Ltd., Shanghai, China. The binding buffer (BB pH 7.4) was applied in all experiments containing 1 mM MgCl_2_, 140 mM NaCl, 2.7 mM KCl, 0.1 mM Na_2_HPO_4_, and 1.8 mM KH_2_PO_4_ [28]. Ultra-pure water was obtained from a Milli-Q water-purification system (Millipore, Bedford, MA, USA). 

An isolate H1 of *Aspergillus niger* isolated from the wine production region in Hexi Corridor, Gansu Province, which was provided by the College of Food Science and Engineering, Gansu Agricultural University, Lanzhou, China. The pathogen was grown on potato dextrose agar (PDA) medium for 7–10 days. 

### 4.2. Apparatus

All the electrochemical experiments were performed with a CHI-600E workstation (Chen Hua Instrument Company, Shanghai, China). A three-electrode configuration was used in the whole experiments, with gold electrode (diameter 2 mm) as working electrode, platinum (Pt) wire as counter electrode, and Ag/AgCl electrode as the reference electrode. All the electrodes were purchased from Chen Hua Instrument Company (Shanghai, China).

### 4.3. Preparation of Gold Electrode

Gold electrode was polished with 0.1 and 0.05 μm alumina powder on microcloth pads, then ultrasonic cleaning with deionized water 3 min after anhydrous ethanol ultrasonic 3 min. This electrode was ultrasonic cleaned in the mixture of concentrated H_2_SO_4_ and 30% H_2_O_2_ solution (v:v, 7:3) for 1 min. After copious rinsing with water, the gold electrode was placed in 0.5 mol/L H_2_SO_4_ solution and potential cycling by cyclic voltammetry in the potential range of −0.2~1.6 V (50 mV/s 10 min) until obtained the cleaning and stable gold surface. 

### 4.4. Preparation of Label-Free Impedimetric Aptasensor

The pretreated gold electrode was immersed in 5 × 10^−3^ mol/L cysteamine solution for 24 h in darkness at 4 °C. The AuNPs/CA/Au modified electrode was obtained by dipping electrode in the gold nanoparticles in darkness. The gold nanoparticles monolayer surface treated with 10 μL of 0.35 μM aptamer in BB solution by drop-coating and incubated for 90 min at 28 °C to prepare the aptamer modified electrode (Apt/AuNPs/CA/Au). The prepared electrode was incubated in 10 μL 1% BSA solution for 60 min. Finally, the label-free impedimetric aptasensor was prepared for direct detection of OTA, stored at 4 °C in dark. When OTA was detected, 10 μL OTA solutions with different concentrations were deposited on the BSA/Apt/AuNPs/CA/Au electrode surface and then incubated for 60 min. After each modification step, the monolayer modified electrode was rinsed with BB and water for 3 min to remove the physical adsorbed substances. Each OTA solution or OTA sample was detected for three times.

### 4.5. Electrochemical Measurements

The potential cycling range of cyclic voltammetry was from −0.2 V to 0.6 V, the scanning rate was 50 mV/s, the measured solution was 0.5 mM K_4_[Fe(CN)_6_]/K_3_[Fe(CN)_6_] (1:1) and 10 mM KCl was used as the supporting electrolytic material. 

Impedimetric detection applied open circuit potential, the AC amplitude was 5 mV and the frequency range were 0.1~10,000 Hz. All the measurements were performed in the 10 mM KCl solution of 0.5 mM K_4_[Fe(CN)_6_]/K_3_[Fe(CN)_6_] (1:1). Impedimetric detection data conform to equivalent circuit of Randles’ model (R(Q(RW))) which has been applied to SAMs (Lu et al., 2010). All date obtained by EIS was analyzed by ZSimpWin electrochemical software (Version3.00, EChem Software, eDAQ Pty Ltd, Sydney, Australia). To eliminate the diversity between different electrodes and to ensure the accuracy of the data, experiment dates were relative normalized according to the following Equation (1) in the literature [27].

(1) $$\Delta R_{ct}=\frac{R_{ct\left(Apt-OTA\right)-}R_{ct\left(bare\;Au\;electrode\right)}}{R_{ct\left(aptamer\right)-}R_{ct\left(bare\;Au\;electrode\right)}}$$

### 4.6. Sample Preparation

Red wine, white wine, red grape juice and purple grape juice were purchased from the local supermarket. The OTA-free grape commodities were spiked with the known concentration of OTA solution samples, and diluted with water to get a series of different concentration of OTA (1 ng/mL, 4 ng/mL, 6 ng/mL).

In order to eliminate the effects of phenolic compounds of the table wine and grape juice, 0.1 g polyvinylpyrrolidone K-25 (PVP, MW = 40,000 g/mol) was added to 10 mL wine or grape juice spiked with OTA [15,41]. After complex precipitation of PVP and phenolic compounds were filtered out, the treated grape commodity samples were diluted, the final concentration of OTA in the samples were 1 ng/mL, 4 ng/mL, 6 ng/mL, respectively.

Centennial seedless and red globe grapes were harvested at commercial maturity from the vineyard of College of Food Science and Engineering, Gansu Agricultural University, and stored at 4 °C. Sterilized grape samples were inoculated with 10 μL spore suspension of *Aspergillus niger* with the concentration of 1 × 10^5^ CFU/mL. The inoculated grapes were incubated for 7 days in the dark at room temperature with the 80–85% relative humidity and then stored at −80 °C until analysis. OTA was extracted from inoculated grape samples according to the following method. 5 g of milled sample was added to 20 mL of a mixture of methanol/water (84:16, v:v) homogenized, followed by centrifugation at 10,000× *g* for 5 min. After filtered, 0.1 g PVP was added to 10 mL supernatant, and then stirred to continue the filtration. The OTA extractive of grape samples was diluted by 10 times for aptasensor detection.

## Figures and Tables

**Table 1:** Comparison of recently reported label-free impedimetric aptasensors for determination of OTA.

Aptasensors	LOD (ng/mL)	Linear Range (ng/mL)	References
Au-ATP-rGO composite modified Au Electrode	0.03	0.1–200	[20]
Thionine and IrO2 NPs modified SPCE	0.0056	0.004–40	[21]
DNA/PANI–SA/ITO modified glass plates	0.1	0.1–10	[26]
Diazonium-coupling reaction modified SPCE	0.15	0.15–2.5	[27]
Thiolated aptamer modified Au electrode	0.05–0.16	0.04–40	[35]
Polypyrrole-dendrimers modified gold surface	0.002	0.002–6	[37]
Layer-by-layer self-assembly modified Au electrode	0.03	0.1–10	This work

**Table 2:** Recovery results of OTA in spiked red win, white wine, red grape juice, and purple grape juice (n = 3).

Sample	OTA Added, ng/mL	OTA Found, ng/mL	RSD (%)	Recovery (%)
Red wine	1	0.98 ± 0.04	3.96%	98.24%
	4	4.17 ± 0.32	7.42%	100.04%
	6	5.51 ± 0.41	1.30%	92.83%
White wine	1	1.01 ± 0.07	6.47%	100.81%
	4	4.17 ± 0.32	7.77%	104.21%
	6	5.51 ± 0.41	7.41%	91.90%
Red grape juice	1	0.98 ± 0.04	1.44%	99.04%
	4	4.00 ± 0.30	6.81%	90.56%
	6	5.57 ± 0.07	8.20%	95.75%
Purple grape juice	1	0.99 ± 0.02	1.90%	93.44%
	4	3.62 ± 0.25	5.78%	97.68%
	6	5.75 ± 0.47	2.56%	92.25%

**Table 3:** Production of OTA in grapes inoculated for different time, centennial seedless, and red globe grapes (n = 3).

Sample	Inoculated Time (day)	OTA Found by Aptasensor (ng/mL)	RSD (%)	OTA Production, (μg/kg)
red globe	0	0.01 ± 0.01	5.81	-
	3	1.56 ± 0.04	2.79	62.40
	5	2.96 ± 0.06	2.11	118.40
	7	5.00 ± 0.12	2.24	200.00
centennial seedless	0	−0.20 ± 0.01	5.10	-
	3	0.50 ± 0.05	10.96	20.00
	5	3.20 ± 0.08	2.39	128.00
	7	3.51 ± 0.15	4.23	140.40
